# Author Correction: Marine phosphorus and atmospheric oxygen were coupled during the Great Oxidation Event

**DOI:** 10.1038/s41467-026-68615-w

**Published:** 2026-01-19

**Authors:** Matthew S. Dodd, Chao Li, Haodong Gu, Zihu Zhang, Mingcai Hou, Aleksey Sadekov, Carlos Alberto Rosière, Franco Pirajno, Lewis Alcott, Frantz Ossa Ossa, Benjamin J. W. Mills, Andrey Bekker

**Affiliations:** 1 School of Earth and Oceans, University of Western Australia, Perth, WA Australia; 2 State Key Laboratory of Oil and Gas Reservoir Geology and Exploitation & Institute of Sedimentary Geology, Chengdu University of Technology, Chengdu, China; 3 International Center for Sedimentary Geochemistry and Bio-geochemistry Research, Chengdu University of Technology, Chengdu, China; 4 State Key Laboratory of Geomicrobiology and Environmental Changes, China University of Geosciences, Wuhan, China; 5 Centre for Microscopy, Characterisation and Analysis, The University of Western Australia, Perth, WA Australia; 6 Department of Geology, Universidade Federal de Minas Gerais, Belo Horizonte, Minas Gerais Brazil; 7 School of Earth and Environment, University of Bristol, Bristol, UK; 8 Department of Earth Sciences, Khalifa University of Science and Technology, Abu Dhabi, UAE; 9 Department of Geology, University of Johan-nesburg, Johannesburg, South Africa; 10 Polar Research Center, Khalifa University of Science and Technology, Abu Dhabi, UAE; 11 School of Earth and Environment, University of Leeds, Leeds, UK; 12 Department of Earth and Planetary Sciences, University of California, Riverside, CA USA

Correction to: *Nature Communications* 10.1038/s41467-025-64194-4, published online 15 October 2025

In the version of the article initially published, the top section of Fig. 1 included a cartoon picture of the fossil *Grypania Spirals* at 2.1 billion years ago. This illustration has since been removed as current literature places the first appearance of this fossil at ca. 1.8 billion years ago. This correction has been made to the HTML and PDF versions of the article.

