# Supplementary material for: Marine phosphorus and atmospheric oxygen were coupled during the Great Oxidation Event
Source: Nat Commun. 2025 Oct 15;16:9151. doi: 10.1038/s41467-025-64194-4 (PMC12528410; doi:10.1038/s41467-025-64194-4)
Supplement: Supplementary file 1 — Supplementary Information [file 41467_2025_64194_MOESM1_ESM.pdf]

Marine phosphorus and atmospheric oxygen were coupled during the Great Oxidation Event

Matthew S. Dodd[1,2,3,4*#], Chao Li[2,3,4*#], Haodong Gu[4], Zihu Zhang[2,3], Mingcai Hou[2], Aleksey Sadekov[1,5], Carlos Alberto Rosière[6], Franco Pirajno[7], Lewis Alcott[8], Frantz Ossa Ossa[8,9,10], Benjamin J. W. Mills[11], Andrey Bekker[9,12]

[1] School of Earth and Oceans, University of Western Australia, 35 Stirling Highway, Perth, 6009, Western Australia, Australia
[2] State Key Laboratory of Oil and Gas Reservoir Geology and Exploitation & Institute of Sedimentary Geology, Chengdu University of Technology, Chengdu 610059, China
[3] International Center for Sedimentary Geochemistry and Biogeochemistry Research, Chengdu University of Technology, Chengdu 610059, China
[4] State Key Laboratory of Geomicrobiology and Environmental Changes, China University of Geosciences, Wuhan 430074, China
[5] Centre for Microscopy, Characterisation and Analysis, The University of Western Australia, Perth, WA 6009, Australia
[6] Department of Geology, Universidade Federal de Minas Gerais, 31270-901 Belo Horizonte, Minas Gerais, Brazil
[7] School of Earth and Environment, University of Bristol, Bristol, BSA 1RJ, UK
[8] Department of Earth Sciences, Khalifa University of Science and Technology, Abu Dhabi, UAE
[9] Department of Geology, University of Johannesburg, Auckland Park, Johannesburg, South Africa
[10] Polar Research Center, Khalifa University of Science and Technology, Abu Dhabi, UAE
[11] School of Earth and Environment, University of Leeds, Leeds LS2 9JT, UK
[12] Department of Earth and Planetary Sciences, University of California, Riverside, California 92521, USA

* Corresponding authors: matthew.dodd@uwa.edu.au, chaoli@cdut.edu.cn
[#] These authors contributed equally

Supplementary Information 1. Study regions and samples in chronological order

Wooly Dolomite, Wyloo Group, Western Australia ca. 2,030 Myr

The Wooly Dolomite of the Wyloo Group in the Horseshoe rift basin of Western Australia contains platform carbonates deposited after the Lomagundi carbon isotope excursion. Samples of the Wooly Dolomite are the same as those analyzed in Bekker et al. (2016). The Wooly Dolomite consists of platform carbonate with interbedded dolostone and siltstone, with dolostone lithofacies, containing stromatolitic breccia and cross-bedding along with clastic fragments with oncolithic overgrowths, interpreted as a high-energy, shallow-marine sequence (Bekker et al., 2016). Age constraints for the Wooly Dolomite come from a SHRIMP U-Pb zircon age of 2,031 ± 6 Myr from volcaniclastic siltstones and sandstones within the Wooly Dolomite (Müller et al., 2005). The Wyloo Group has been metamorphosed to the lower greenschist facies, prehnite-pumpellyite subfacies (Seymour et al., 1988).

Nash Fork Formation, Snowy Pass Supergroup, Wyoming, USA, ca. 2,200-2,050 Myr

The Nash Fork Formation overlies the Sugarloaf Quartzite and is the lowermost unit in the upper Libby Creek Group from the Snowy Pass Supergroup in the Medicine Bow Mountains, Wyoming (Bekker et al., 2003). The Snowy Pass Supergroup forms broad open folds; the degree of metamorphism is greenschist facies, biotite grade (Houston et al., 1981). The Nash Fork Formation is 1.7 km thick and is separated into lower, middle, and upper parts by two carbonaceous shales. The lower Nash Fork Formation consists of interlayered massive and stromatolitic dolostone facies, heterolithic siliciclastic-carbonate facies, and nodular dolostone facies associations, interpreted to represent deposition in upper intertidal, supratidal, and peritidal settings (Bekker et al., 2003). Ubiquitous sulfate molds in argillites and dolostones suggest an arid depositional environment. The middle Nash Fork Formation consists of three members and includes carbonaceous shales and heterolithic siliciclastic-nodular dolostone facies. Carbonaceous shale facies consist of black, organic-rich shale with pyrite, siltstone, and sandstone and likely developed in response to platform drowning. The upper Nash Fork Formation, above the upper carbonaceous shale, consists of a massive dolostone facies and contains a prominent karstic surface overlain by fluvial quartzite, dividing the member into two parts. This facies consists of upward-shallowing parasequences that reflects deposition in outer shelf to subaerial environments. There is no evidence for a restricted depositional setting for the Nash Fork Formation in the form of such features as bedded evaporites. Rather, the presence of facies indicative of tidal flat, lagoonal, inner and outer shelf, and drowned platform environments as well as the sequence architecture (Bekker and Eriksson, 2003) suggest that the Nash Fork Formation developed entirely in open-marine settings on a passive margin along the southern edge of the Wyoming Craton. Samples for this study are the same as those in Bekker et al., 2003. The age of the succession is constrained by U- Pb ages of detrital zircons from the base of the succession (Magnolia Formation) in the Sierra Madre at 2451± 9 Myr (Premo and Van Schmus, 1989) and a minimum age for the Nash Fork Formation is constrained by the younger mafic volcanism that has been dated elsewhere on the Wyoming craton to approximately 2,000 – 1,970 Myr (Cox et al., 2000; Redden et al., 1990). Recent work suggests a maximum age for the Nash Fork formation of 2118 ±

2.4 Myr (Mammone et al., 2022). Combined, these age constraints suggest that the Nash Fork Formation was deposited between ca. 2,118 and 1,970 Myr. The Nash Fork Formation is younger than the Paleoproterozoic glacial epoch as it overlies thick, mature, Al-rich quartzites in the Snowy Pass Supergroup, correlative to those above the three glacial diamictites of the Huronian Supergroups (Bekker et al., 2003). Additionally, the highly positive $\delta^{13}C_{carb}$ values (>20‰) in the basal Nash Fork Formation are similar to values (>18‰) for the Lomagundi Excursion in Karelia, Russia. Considering the aforementioned information, the boundary between the upper and lower Nash Fork formations is inferred to correspond to the end of the Lomagundi carbon isotope excursion, providing a ca. 2,200 - 2,060 Myr age bracket for the Nash Fork Formation (Bekker et al., 2003).

FC Formation, Francevillian Group, Gabon, ca. 2,220-2,090 Myr
The Francevillian Group marine sedimentary rocks are exquisitely preserved within the Francevillian basin, Gabon. The Francevillian basin consists of unmetamorphosed sedimentary rocks (Gauthier-Lafaye and Weber, 1989), with the preserved early diagenetic smectite/illite indicating only a moderate degree of diagenesis (Ossa Ossa et al., 2013). The group was deposited during the Paleoproterozoic in a foreland basin setting and is subdivided into four lithostratigraphic units, FA to FD formations, which rest unconformably on the Archean basement rocks (Weber, 1968). The FA Formation consists of mainly fluviatile and deltaic sandstones. The FB Formation consists of marine sediments deposited mainly below storm wave-base and includes interlayered shales, sandstones, and conglomerates, fining upwards to predominantly shales with a thin iron formation overlain by black shales and a thick interval rich in manganese. The overlying FC Formation is dominated by dolostones and stromatolitic cherts, indicating shallow-water conditions. Stromatolites are found on topographic highs at the base of the FC Formation (Bertrand-Sarfati and Potin, 1994). The FD Formation corresponds to black shales deposited during a transgressive phase. Diagenetic illites from the top of the FB_{1b} submember yielded a Sm-Nd age of 2,099 ± 115 Myr (Bros et al., 1992), while a more precise U-Pb zircon age of 2,083 ± 6 Myr was reported from a welded tuff near the top of the FD Formation (Horie et al., 2005).

Juderina Formation, Yerrida Group, Western Australia, ca. 2,100 Myr
The Juderina Formation is up to 1000 m thick and comprises mainly cross-bedded and parallel-laminated quartz sandstone, siltstone, and conglomerate layers (Pirajno et al., 1998). The Juderina Formation consists of a basal unit called the Finlayson Member that lies nonconformably on the Archean basement with a gentle northward dip (Pirajno et al., 1998). Above the Finlayson Member, a sequence (up to 160 m thick) of interbedded evaporites and coastal stromatolites defines the Bubble Well Member (Pirajno et al., 1998). The remainder of the Juderina Formation records a transition from a coastal/shallow-marine to subtidal environment (Pirajno et al., 1998). The Juderina Formation has a transitional contact with the Johnson Cairn Formation, which is up to 1250 m thick and consists of pyritic, parallel, and finely-laminated black mudstone and iron-rich shale, with subordinate graded silty layers and thin dolostone bands. A depositional age of 2173 ± 80 Ma is based on a Pb–Pb isochron age for stromatolitic carbonates of the Bubble Well Member (Woodhead and Hergt, 1997), which is supported by the maximum depositional age based on detrital zircons (Sheppard et al., 2016). Very low-grade metamorphism of the Juderina Formation, which corresponds to anchimetamorphism (Kisch, 1987), is

constrained by the incipient development of illite, but the general preservation of original detrital clays. This very low metamorphic grade is confirmed by the preservation of primary mineral assemblages in associated mafic units (Adamides et al., 1999). Samples include 5 hand samples collected from outcrops at the Eagle Roost section and 18 samples from drill core THD-001, which was drilled at 25°24'13.32"S; 119°43'35.76"E.

Silverton Formation, upper Pretoria Group, South Africa, ca. 2,100 Myr
The Silverton Formation belongs to the Paleoproterozoic Pretoria Group deposited in the open-marine Transvaal basin of South Africa. In our study area, in the northwestern part of the Transvaal basin, the Silverton Formation experienced only lower greenschist facies metamorphism. Samples were collected in South Africa 1 to 2 km from the border with Botswana. The deltaic to offshore marine Silverton Formation is sandwiched between shallow-marine, tidally-influenced quartz sandstones. The Silverton Formation contains carbonates, interlayered with shales that are partially dolomitized, laminated, and rarely display the features of a shallow-water depositional environment such as wave ripples, domal stromatolites, silicified nodules, soft-sediment deformation structures, water and gas escape structures, and small-scale cross-bedding (Bekker et al., 2008). The formation is bracketed in age by the underlying ca. 2,220 Myr Hekpoort Lava and the overlying or intruding 2,060–2,050 Myr Rooiberg Felsite Group and Bushveld Complex, respectively (Buick et al., 2001; Dorland, 2004; Walraven, 1997). Further indirect age constraints might be inferred from the ca. 2,140 – 2,120 Myr U–Pb ages of authigenic metamorphic monazite in metamorphic rocks of the Chuniespoort Group and the older Central Rand Group of the Witwatersrand Supergroup, reflecting a tectonic event in the Transvaal basin (Rasmussen et al., 2007), likely corresponding to the transition from the passive continental margin to the foreland basin at the stratigraphic level of the Silverton Formation (Bekker et al., 2008).

Fecho do Funil Formation, Minas Group, Brazil, ca. 2,100 Myr
The Fecho do Funil Formation contains phyllite, siltstone, ferruginous quartz arenite, and lenses of argillaceous dolostone. Phyllite is more abundant in the lower part of the formation and dolostone content increases up section. Dolostone lenses are up to 30 meters thick, and contain long, columnar and domal stromatolites (Dardenne and Campos Neto, 1975; Garcia et al., 1988), and lack siliciclastic detritus, indicating a subtidal to intertidal depositional setting. A 2110 ± 110 Ma Pb-Pb carbonate date of the Fecho do Funil Formation provide a minimum age for the unit (Babinski et al., 1995). The Minas Supergroup experienced regional metamorphism at low-grade greenschist facies based on mineral assemblages (Herz, 1978).

Mcheka Formation, Lomagundi Group, Zimbabwe, ca. 2,150 Myr
The Mcheka Formation is the lowermost unit of the Lomagundi Group (Tennick and Phaup, 1976). It unconformably overlies the Deweras Group. It comprises basal pebbly grits overlain by the Lower Dolomite, Phyllite, Quartzite, Upper Dolomite, and Sandy Argillite members. The Lower Dolomite consists of whitish-pink mottled dolostone, with thin argillaceous, arenaceous interbeds and small domical stromatolites (Stowe, 1978). It also contains centimetre-sized, sparry calcite nodules, and fan-shaped carbonate pseudomorphs, possibly after sulphate evaporites (Master and Verhagen, 1998). The Upper Dolomite shows a greater variation in texture, crystallinity, and colour, and contains bands of biotite-rich phyllite, sericitic

and feldspathic grits, and chloritic quartzite. It also contains two bands of stromatolites, which include both domical and columnar varieties (Stowe, 1978; Tennick and Phaup, 1976). An oolitic horizon is present in the upper dolomite (Stowe, 1978). The metamorphic grade of the Mcheka Formation is lower greenschist facies, quartz–muscovite–chlorite–albite subfacies (Master et al., 2010). The Lomagundi Group dolostones have been dated directly using the Pb-Pb technique at 2150 ± 50 Myr (Schidlowski and Todt, 1998).

Duitschland Formation, South Africa, ca. 2,400 Myr

The Duitschland Formation sits at the base of the Pretoria Group in the Transvaal basin. The depositional age of the Duitschland Formation is constrained to be between ~2480 and ~2310 Ma based on 2,480 ± 6 Myr U-Pb SHRIMP age for the underlying Penge Iron Formation and a 2307 ± 8 Myr age of tuff layers in the overlying Timeball Hill Formation, respectively (Nelson et al., 1999; Rasmussen et al., 2013). Detrital zircon geochronology suggests a maximum depositional age of 2424 ± 12 Ma for the upper Duitschland Formation (Schröder et al., 2016). Recently, the upper Duitschland Formation was dated with the Re-Os method at 2443 ± 33 Ma (Millikin et al., 2024). The lower part of the Duitschland Formation begins with conglomerate and a glacial diamictite overlain by thick, finely-laminated carbonaceous shale, which is in turn overlain by interbedded layers of limestone, marl, and breccia. Carbonate layers are thin and composed of grey, finely-laminated limestone rhythmites, likely deposited in a deep-water setting, below a storm wave-base. Chert and limestone breccias in the lower part of the succession are interpreted as a gravity-flow slope deposit (Swart, 1999). The upper half of the formation consists of fine- to coarse-grained quartzite, conglomerates, shale, and carbonate. An up section decrease in the abundance of shale and the presence of ripple marks in a fine-grained quartzite suggest that deposition occurred in a shallow-water environment. The first thick carbonate is limestone composed of isopachous sheet cements, likely formed as syn-sedimentary cements on the seafloor (Hoffman, 1975; Kah and Knoll, 1996). The upper part of the Duitschland Formation is intruded by two sills related to the nearby Bushveld Complex, which produced contact metamorphism of some carbonates in this part of the section at around 450-475°C to form hornfels (Warke, 2017). Above the first sill is a shallow-water limestone with chert nodules and large stromatolitic domes (Martini, 1979). The overlying dolostone layer is also stromatolitic near its base and contains oolites and fan-shaped chert nodules as well as ripple marks with flaser, muddy laminations above. The uppermost dolostone beds contain domal stromatolites as well as silicified giant ooids (up to 0.5 cm) and grapestones, suggesting a marine depositional environment. The Duitschland Formation is only locally preserved in the northeastern part of the Transvaal basin, however a marine depositional environment for this succession is inferred based on sedimentologic evidence such as giant spherical ooids and grapestones of the upper Duitschland Formation, which strongly resemble those found in the dominantly marine Neoproterozoic successions (Swett and Knoll, 1989).

Kona Dolomite, Marquette Range Supergroup, Michigan, USA, ca. 2,170 Myr

The Kona Dolomite of the Marquette Range Supergroup sits within the Chocolay Group above the Mesnard Quartzite and the underlying glacially-influenced Enchantment Lake Formation (Bekker et al., 2006). The age of the Enchantment

Lake Formation is bracketed by the age of the youngest detrital zircon of 2,288 ± 15 Ma and the age of the oldest hydrothermal xenotime of 2,133 ± 11 Ma (Vallini et al., 2006); the overlying Wewe Slate contains tuffs dated at 2174 ± 9 and 2172 ± 6 Ma (Rasmussen et al., 2024). The Kona Dolomite was metamorphosed to lower greenschist facies. The Chocolay Group was likely deposited in a failed rift basin extending to the west from the open continental margin where the upper part of the Huronian Supergroup accumulated (Young, 1983).

Saunders Formation, Marquette Range Supergroup, Wisconsin, USA, ca. 2,300–2,140 Myr

The metamorphic grade of the Saunders Formation of the Iron River-Crystal Falls district in Wisconsin ranges from greenschist to amphibolite facies (James, 1955) and it consists of dolostones with variable amounts of siliciclastics (Larue, 1979). The Saunders Formation consists of cherty dolostone, massive white and pink finely crystalline dolostone with stromatolites, sandy dolostone, and impure calcareous shales (Allen, 1910). Samples were collected from two areas to the south of the Brule River (see Bekker et al., 2006). Based on marked difference in $\delta^{13}C_{carb}$ composition, basin analysis, and detrital zircon and xenotime ages for the conformably underlying Sturgeon Quartzite (2302 ± 4 Ma and 2115 ± 5 Ma, respectively; Vallini et al., 2006), it has been suggested that Saunders Formation is slightly older than the Kona Dolomite and was deposited before the Lomagundi Excursion (Bekker et al., 2006; Rasmussen et al., 2024).

Gordon Lake Formation, Upper Huronian Supergroup, Ontario, Canada, ca. 2,310 Myr

The Gordon Lake Formation is 300–700 m thick and consists of well-bedded, variegated mudstone and siltstone, chert, and minor, fine-grained quartz sandstone that contains rare beds and nodules of dolostone (Hofmann et al. 1980; Bennett et al. 1991; Young 1991; Jackson 1994; Bekker et al. 2006). The unit is subdivided into three members: a lower member consisting of red sandstone and siltstone with chert and anhydrite/gypsum nodules (Chandler 1988); a middle member composed of green siltstone and mudstone with minor sandstone; and an upper member made up of red siltstone, mudstone, and chert. Chamosite and glauconite are present in the middle part of the formation indicating marine environment (Wood 1973; Chandler 1986). The lower part of the formation was deposited in a sabkha tidal-flat setting; the middle part represents transgressive storm-influenced marine environment below tidal current influence; and the upper part is the regressive section grading towards tidal-flat setting of the overlying Bar River Formation (Chandler 1986). Carbonates of the Gordon Lake Formation are restricted to the base of the formation and occur in several localities (Bekker et al., 2006). Thin laminated dolostone beds with fenestral fabrics were sampled at Plummer Township (Hofmann et al. 1980). The most extensive outcrop of the dolostone unit is in Fenwick Township and consists of pink dolomicrite, and pale-grey doloarenite with quartz grains, oolites, and intraformational flat-pebble conglomerate. The carbonate unit is at least 30 m thick in this area (Bennett et al. 1989). The age of the Gordon Lake Formation is constrained at 2318 ± 8 Ma, based on the U-Pb SHRIMP zircon age of tuff layers (Rasmussen et al., 2013, 2024). The Huronian Supergroup has been subjected to lower greenschist facies metamorphism (Card, 1978).

Espanola Formation, Lower Huronian Supergroup, Ontario, Canada, ca. 2,400 Myr

The Espanola Formation is the only thick and extensive carbonate unit of the Huronian Supergroup, which occurs directly above glacial diamictite of the Bruce Formation and is overlain by fluvial sandstones of the Serpent Formation. Interlayered mafic volcanic rocks and intrusive contacts with the Murray and Creighton granites constrain the age of the basal Huronian Supergroup to 2,490–2,450 Myr (Krogh et al. 1984, 1996; Ketchum et al., 2013; Bleeker et al., 2015). Based on this maximum depositional age and the 2.31 Ga age of the Gordon Lake Formation in the Upper Huronian Supergroup, the age of the Espanola Formation is estimated to be around 2,400 Myr (Bekker et al., 2005). The lowermost unit of the Espanola Formation consists of a thin-bedded grey siltstone and recrystallized limestone with 1.5–3.0 m thick shale at the base. Limestone beds are thinly laminated and contain soft-sediment deformation structures. Stromatolites are conspicuously missing in the Espanola Formation, with the exception of one locality (Hoffmann et al. 1980). The dolostone member consists of interlayered ferruginous dolostone, calcareous siltstone, and limestone. The uppermost unit contains mudcracks, symmetrical and ladder ripples, and wave ripples indicating a shallow-water depositional setting (Bekker et al., 2005). Samples were collected from the S-54 and Kerr-McGee Corp. 150/1 drill-holes.

Gandarela Formation, Minas Group, Brazil, ca. 2,430 Myr

The Gandarela Formation has a gradational contact with the Caûe Iron Formation and includes dolostones, limestones, dolomitic phyllite, a dolomitic iron formation, and phyllite (Dorr, 1969). Carbonates in the middle part of the Gandarela Formation contain well preserved stromatolites and oncolites (Souza and Muller, 1984), indicating deposition in high-energy intertidal to shallow subtidal environments. Intraformational dolostone and chert flat-pebble conglomerates, which range in thickness from few centimetres to more than 1 meter, occur in the upper part of this unit (Dorr, 1969). A minimum age constraint for the formation is provided by a 2420 ± 19 Ma Pb-Pb carbonate age (Babinski et al., 1995).

Carawine Dolomite, Hamersley Group, Western Australia, ca. 2,630 Myr

The Carawine Dolomite is a part of the Hamersley Group in Western Australia and contains stromatolites, oolites, pisolites, oncolites, evaporite facies, and wave ripples, indicating deposition on a shallow-marine carbonate platform within the photic zone with episodic exposure (Simonson et al., 1993). This depositional environment is similar to those environments, which preserve highly positive $\delta^{13}C_{carb}$ values of the Lomagundi Excursion, making it an excellent comparative unit. The age of the Carawine Dolomite is 2630 ± 6 Ma, based on the U-Pb SHRIMP zircon age for tuff beds (Rasmussen et al., 2005). The formation was metamorphosed to the prehnite–pumpellyite grade (Smith et al., 1982). The Carawine Dolomite samples analysed in this study are from the drill hole DDH8BG5, which was drilled at 22°15’S; 120°36’E and is currently stored at the Perth core library.

Supplementary Information 2: Discussion on the validity of the CAP data interpretation

The positive correlation between CAP and $\delta^{13}C_{carb}$ values seen in this study dataset is inverse compared to previously seen relationship between authigenic carbonate

$\delta^{13}C_{carb}$ values and CAP (Dodd et al., 2021). Given that pore water P and $\delta^{13}C_{DIC}$ values are generally negatively correlated (aside from the methanic window), a positive correlation of $\delta^{13}C_{carb}$ values and CAP concentrations suggests that organic matter remineralisation in carbonate sediments is unlikely to be an overarching control on the observed relationship between $\delta^{13}C_{carb}$ values and CAP concentration in this study. Similarly, changes in CO_3^{2-} concentration in pore fluids resulting from carbonate dissolution or organic matter remineralisation are unlikely to account for the relative difference in CAP values between low and high $\delta^{13}C_{carb}$ carbonates. This is because experimental work shows changes in CAP values resulting from changes in dissolved CO_3^{2-} concentration are smaller than those observed between the samples (Dodd et al., 2023).

Carbonate trace element data is often used to screen for significant diagenetic alteration of carbonates. Commonly, Mn/ Sr is used, which has been shown to correlate with the degree of alteration based on $\delta^{44}Ca_{carb}$ analyses of carbonates from modern platforms (Higgins et al., 2018). Similarly, CAP and Mn/Sr show negative correlation on modern carbonate platforms. Additionally, metamorphic alteration induce correlation between CAP and $\delta^{18}O_{carb}$ values. There is however a positive correlation of $\delta^{13}C_{carb}$ and $\delta^{18}O_{carb}$, and CAP and $\delta^{18}O_{carb}$, across our sample set (Extended Data Fig. 1; Table 1), which might be evidence for diagenetic (Brand and Veizer, 1981), evaporitic (Horton et al., 2016), or metamorphic controls on CAP concentration. However, such correlations are common to much younger and well-preserved Phanerozoic carbon isotope excursions and may record primary environmental perturbations to the Earth system (Stanley, 2010). Therefore, correlation of $\delta^{13}C_{carb}$ and $\delta^{18}O_{carb}$ is not diagnostic indicator of carbonate origin.

Lastly, CAP distribution coefficients are known to vary among carbonate minerals, such as aragonite, dolomite and calcite, which could result in mineralogical control on the observed CAP values and trends. This is clearly noticeable for the Espanola and Ganderela formations where CAP values are on average 4.6 and 1.9 times higher in dolomite than calcite, respectively. However, for the Silverton Formation CAP values in calcite are of larger than those in dolomite samples outside of the LE, suggesting that elevated CAP concentration during the LE is not a result of mineralogical control. Moreover, the majority of samples in this study are dolostones, therefore the overarching correlation of $\delta^{13}C_{carb}$ and CAP is defined by dolomite minerals with minimal data points (<10%) from calcite.

Independent validation of carbonate and CAP preservation

An independent test on the fidelity of the CAP records is the comparison of sulphur isotopic composition ($\delta^{34}S$) of sulphate in evaporites and carbonate-associated sulphate. Previous studies have shown that $\delta^{34}S_{SO4}$ values in both evaporite and carbonate minerals in the formations analysed in this study (Gordon Lake Formation, Kona Dolomite, and Silverton and Mcheka formations) are within error of one another across the GOE interval (Planavsky et al., 2012). Given that $\delta^{34}S_{SO4}$ values in sulphate evaporite and carbonate minerals are independent records of seawater $\delta^{34}S_{SO4}$ and preserve similar values, we can confidently assess that carbonate chemistry is faithfully capturing seawater chemistry. Moreover, diagenetic fluid-rock alteration models predict that under conditions typical of marine diagenesis, CAP is more resilient than CAS (Dodd et al., 2023). Consequently, the preservation of seawater $\delta^{34}S_{SO4}$ values in the analysed carbonates supports a primary origin for the measured CAP values.

Correlation of CAP with lithological changes

We note that in the case of the Francevillian FC Formation CAP values decline following a transition from low to high Mn-carbonate, which might be indicative of carbonate and CAP alteration (Mayika et al., 2020). While plausible, CAP alteration to lower values in this high-Mn carbonate is unlikely for two reasons: 1) high-Mn carbonate is generally associated with reducing water-column conditions under which dissolved P level is likely to be elevated not depleted, and 2) this transition to high-Mn carbonate and lower CAP values is not shown by any other formation in our study where CAP and $\delta^{13}C_{carb}$ co-vary.

Lastly, we note that while stratigraphic correlation of CAP and $\delta^{13}C_{carb}$ provides one line of evidence for positively coupled trends, the overarching trend of higher CAP in carbonate with higher $\delta^{13}C_{carb}$ values comes from multiple independent formations, capturing a diversity of lithological characteristics from multiple time periods. For example, high CAP and $\delta^{13}C_{carb}$ values are found in sediments from both intertidal evaporitic regimes and open-marine settings, which innately would have experienced very different diagenetic histories (e.g., Juderina vs. Silverton formations). Therefore, diagenetic processes linked to changes at lithological boundary at one locality in time and space cannot explain the correlation of CAP concentrations and $\delta^{13}C_{carb}$ values across the breadth of the GOE interval.

Spatial heterogeneity of seawater phosphate concentrations

There are large differences in seawater P concentrations both laterally and vertically in the modern ocean due to ocean circulation and landmass configuration. As a result, changes in ocean circulation, continental landmasses, and sea level could change local P concentrations and, therefore, CAP values. For example, a sea-level rise could produce a lithological facies correlation with CAP values at a given locality. This is however unlikely to explain the positive correlation between CAP and $\delta^{13}C_{carb}$ in this study because 1) in the modern ocean P and $\delta^{13}C_{DIC}$ values are inversely correlated, and 2) CAP values in this study are elevated in shallow-marine settings where P concentrations are theoretically at their lowest due to biological utilisation. There is a possibility that the samples analysed in this study have come from locations not representative of the global ocean, but rather from zones of upwelling or P hotspots in the ocean and vice versa, which could give the illusion of global seawater changes in CAP and $\delta^{13}C_{carb}$. This possibility would however have to reconcile the consistently elevated CAP and $\delta^{13}C_{carb}$ values in markedly different oceanographic settings across both shallow-marine and sometimes evaporitic settings (Nash Fork, Mcheka, Francevillian FC, Fecho do Funil, and Juderina formations) and deep-marine (Silverton Formation) sediments during the Lomagundi Excursion.

References:

Adamides, N. G., Pirajno, F., and Farrell, T. R., 1999, Geology of the CUNYU 1:100 000 sheet: Geological Survey of Western Australia

Alcott, L. J., Mills, B. J. W., and Poulton, S. W., 2019, Stepwise Earth oxygenation is an inherent property of global biogeochemical cycling: Science, v. 366, no. 6471, p. 1333-1337.

Alcott, L. J., Walton, C., Planavsky, N. J., Shorttle, O., and Mills, B. J. W., 2024, Crustal carbonate build-up as a driver for Earth's oxygenation: Nature Geoscience, v. 17, no. 5, p. 458-464.

Allen, R. C., 1910, The Iron River iron-bearing district of Michigan; Publication 3, *in* Survey, M. G. a. B., ed., Volume Geological. Series 2, Michigan Geological and Biological Survey, p. 151.

Babinski, M., Chemale, F., and Van Schmus, W. R., 1995, The Pb/Pb age of the Minas Supergroup carbonate rocks, Quadrilátero Ferrífero, Brazil: Precambrian Research, v. 72, no. 3, p. 235-245.

Bekker, A., and Eriksson, K. A., 2003, A Paleoproterozoic drowned carbonate platform on the southeastern margin of the Wyoming Craton: a record of the Kenorland breakup: Precambrian Research, v. 120, no. 3, p. 327-364.

Bekker, A., Holmden, C., Beukes, N. J., Kenig, F., Eglinton, B., and Patterson, W. P., 2008, Fractionation between inorganic and organic carbon during the Lomagundi (2.22–2.1 Ga) carbon isotope excursion: Earth and Planetary Science Letters, v. 271, no. 1, p. 278-291.

Bekker, A., Karhu, J. A., Eriksson, K. A., and Kaufman, A. J., 2003, Chemostratigraphy of Paleoproterozoic carbonate successions of the Wyoming Craton: tectonic forcing of biogeochemical change?: Precambrian Research, v. 120, no. 3, p. 279-325.

Bekker, A., Karhu, J. A., and Kaufman, A. J., 2006, Carbon isotope record for the onset of the Lomagundi carbon isotope excursion in the Great Lakes area, North America: Precambrian Research, v. 148, no. 1, p. 145-180.

Bertrand-Sarfati, J., and Potin, B., 1994, Microfossiliferous cherty stromatolites in the 2000 Ma Franceville group, Gabon: Precambrian research, v. 65, no. 1-4, p. 341-356.

Blättler, C. L., Kump, L. R., Fischer, W. W., Paris, G., Kasbohm, J. J., and Higgins, J. A., 2016, Constraints on ocean carbonate chemistry and pCO2 in the Archaean and Palaeoproterozoic: Nature Geoscience, v. 10, no. 1, p. 41-45.

Brand, U., and Veizer, J., 1981, Chemical diagenesis of a multicomponent carbonate system; 2, Stable isotopes: Journal of Sedimentary Research, v. 51, no. 3, p. 987-997.

Broecker, W. S., and Maier-Reimer, E., 1992, The influence of air and sea exchange on the carbon isotope distribution in the sea: Global Biogeochemical Cycles, v. 6, no. 3, p. 315-320.

Broecker, W. S., and Peng, T., 1982, Tracers in the sea, Columbia University, New York, Lamont-Doherty Geological Observatory

Bros, R., Stille, P., Gauthier-Lafaye, F., Weber, F., and Clauer, N., 1992, Sm-Nd isotopic dating of Proterozoic clay material: An example from the Francevillian sedimentary series, Gabon: Earth and Planetary Science Letters, v. 113, no. 1-2, p. 207-218.

Buick, I. S., Maas, R., and Gibson, R., 2001, Precise U–Pb titanite age constraints on the emplacement of the Bushveld Complex, South Africa: Journal of the Geological Society, v. 158, no. 1, p. 3-6.

Card, K. D., 1978, Geology of the Sudbury-Manitoulin area, districts of Sudbury and Manitoulin.

Cox, D. M., Frost, C. D., and Chamberlain, K. R., 2000, 2.01-Ga Kennedy dike swarm, southeastern Wyoming : Record of a rifted margin along the southern Wyoming province: Rocky Mountain Geology, v. 35, no. 1, p. 7-30.

Dardenne, M. A., and Campos Neto, M. C., 1975, Estromatolitos colunares na Serie Minas (MG):: Revista Brasileira de Geociencias, v. 5, no. 2, p. 99-105.

Dodd, M. S., Shi, W., Li, C., Zhang, Z., Cheng, M., Gu, H., Hardisty, D. S., Loyd, S. J., Wallace, M. W., vS. Hood, A., Lamothe, K., Mills, B. J. W., Poulton, S. W., and Lyons, T. W., 2023, Uncovering the Ediacaran phosphorus cycle: Nature, v. 618, no. 7967, p. 974-980.

Dodd, M. S., Zhang, Z., Li, C., Algeo, T. J., Lyons, T. W., Hardisty, D. S., Loyd, S. J., Meyer, D. L., Gill, B. C., Shi, W., and Wang, W., 2021, Development of carbonate-associated phosphate (CAP) as a proxy for reconstructing ancient ocean phosphate levels: Geochimica et Cosmochimica Acta, v. 301, p. 48-69.

Dorland, H. C., 2004, Provenance Ages and Timing of Sedimentation of Selected Neoarchean and Paleoproterozoic Successions on the Kaapvaal Craton, Rand Afrikaans University.

Dorr, J. V. N., 1969, Physiographic, stratigraphic, and structural development of the Quadrilatero Ferrifero, Minas Gerais, Brazil, 641A.

Elser, J. J., Dobberfuhl, D. R., MacKay, N. A., and Schampel, J. H., 1996, Organism Size, Life History, and N:P Stoichiometry: Toward a unified view of cellular and ecosystem processes: BioScience, v. 46, no. 9, p. 674-684.

Garcia, A. J. V., Fonseca, M. A., Bernardi, A. V., and Januzzi, A., 1988, Contribuicao ao reconhecimento dos paleoambientes deposicionais do grupo Piracicaba na regiao de Dom Bosco - SW de Ouro Preto, Quadrilatero Ferrıfero-MG: Acta Geologica Leopoldensia, v. 11, no. 27, p. 83-108.

Gauthier-Lafaye, F., and Weber, F., 1989, The Francevillian (Lower Proterozoic) uranium ore deposits of Gabon: Economic Geology, v. 84, no. 8, p. 2267-2285.

Herz, N., 1978, Metamorphic rocks of the Quadrilatero Ferrifero, Minas Gerais, Brazil, 641C.

Higgins, J. A., Blättler, C. L., Lundstrom, E. A., Santiago-Ramos, D. P., Akhtar, A. A., Crüger Ahm, A. S., Bialik, O., Holmden, C., Bradbury, H., Murray, S. T., and Swart, P. K., 2018, Mineralogy, early marine diagenesis, and the chemistry of shallow-water carbonate sediments: Geochimica et Cosmochimica Acta, v. 220, p. 512-534.

Hodgskiss, M. S. W., and Sperling, E. A., 2021, A prolonged, two-step oxygenation of Earth's early atmosphere: Support from confidence intervals: Geology, v. 50, no. 2, p. 158-162.

Hoffman, P., Shoaling-Upward Shale-to-Dolomite Cycles in the Rocknest Formation (Lower Proterozoic), Northwest Territories, Canada, *in* Proceedings Tidal Deposits, Berlin, Heidelberg, 1975// 1975, Springer Berlin Heidelberg, p. 257-265.

Horie, K., Hidaka, H., and Gauthier-Lafaye, F., U-Pb geochronolog and geochemistry of zircon from the Franceville series at Bidoudouma, Gabon, *in* Proceedings 15th Annual Goldschmidt Conference.2005.

Horton, T. W., Defliese, W. F., Tripati, A. K., and Oze, C., 2016, Evaporation induced 18O and 13C enrichment in lake systems: A global perspective on hydrologic balance effects: Quaternary Science Reviews, v. 131, p. 365-379.

Houston, R. S., Lanthier, L. R., Karlstrom, K. E., and Sylvester, G., 1981, Paleoproterozoic diamictite of southern Wyoming, *in* Hambrey, M. J., and Harland, W. B., eds., Earth's Pre-Pleistocene Glacial Record: New York, Cambridge University Press, p. 795-799.
James, H. L., 1955, Zones of regional metamorphism in the Precambrian of northern Michigan: Geological Society of America Bulletin, v. 66, no. 12, p. 1455-1488.
Kah, L. C., and Knoll, A. H., 1996, Microbenthic distribution of Proterozoic tidal flats: environmental and taphonomic considerations: Geology, v. 24, no. 1, p. 79-82.
Kisch, H., 1987, Correlation between indicators ofvery lowgrade metamorphism: in Low Temperature Metamorphism, M, Blackie and Sons, Glasgow.
Larue, D. K., 1979, Sedimentary history prior to chemical iron sedimentation of the Precambrian X Chocolay and Menominee groups (Lake Superior Region) [Ph.D.: Northwestern University.
Mammone, N., Bekker, A., Chamberlain, K., and Kuznetsov, A. B., 2022, Testing the early Paleoproterozoic connection of the Superior and Wyoming cratons with geochronology and geochemistry: Precambrian Research, v. 381, p. 106818.
Martini, J. E. J., 1979, A copper-bearing bed in the Pretoria Group in Northeastern Transvaal: Geological Society of South Africa, v. 6, p. 65-72.
Master, S., Bekker, A., and Hofmann, A., 2010, A review of the stratigraphy and geological setting of the Palaeoproterozoic Magondi Supergroup, Zimbabwe – Type locality for the Lomagundi carbon isotope excursion: Precambrian Research, v. 182, no. 4, p. 254-273.
Master, S., and Verhagen, B. T., 1998, Carbon and oxygen isotopic profile through the high-13C Palaeoproterozoic Lomagundi Dolomite, Magondi Supergroup, Zimbabwe.: China Science Bulletin v. 88.
Mayika, K. B., Moussavou, M., Prave, A. R., Lepland, A., Mbina, M., and Kirsimäe, K., 2020, The Paleoproterozoic Francevillian succession of Gabon and the Lomagundi-Jatuli event: Geology, v. 48, no. 11, p. 1099-1104.
Millikin, A.E.G., Uveges, B.T., Izon, G., Bauer, A.M., Summons, R.E., Evans, D.A.D., and Rooney, A.D., 2024, A new Re-Os age constraint informs the dynamics of the Great Oxidation Event: Geology, v. 52, p. 857–862, https://doi .org /10 .1130 /G52481 .1.
Nelson, D. R., Trendall, A. F., and Altermann, W., 1999, Chronological correlations between the Pilbara and Kaapvaal cratons: Precambrian Research, v. 97, no. 3, p. 165-189.
Ossa Ossa, F., El Albani, A., Hofmann, A., Bekker, A., Gauthier-Lafaye, F., Pambo, F., Meunier, A., Fontaine, C., Boulvais, P., Pierson-Wickmann, A.-C., Cavalazzi, B., and Macchiarelli, R., 2013, Exceptional preservation of expandable clay minerals in the ca. 2.1Ga black shales of the Francevillian basin, Gabon and its implication for atmospheric oxygen accumulation: Chemical Geology, v. 362, p. 181-192.
Pirajno, F., Occhipinti, S. A., and Swager, C. P., 1998, Geology and tectonic evolution of the Palaeoproterozoic Bryah, Padbury and Yerrida Basins (formerly Glengarry Basin), Western Australia: implications for the history of the south-central Capricorn Orogen: Precambrian Research, v. 90, no. 3, p. 119-140.
Planavsky, N. J., 2014, The elements of marine life: Nature Geoscience, v. 7, no. 12, p. 855-856.
Planavsky, N. J., Bekker, A., Hofmann, A., Owens, J. D., and Lyons, T. W., 2012, Sulfur record of rising and falling marine oxygen and sulfate levels during the Lomagundi event: Proceedings of the National Academy of Sciences, v. 109, no. 45, p. 18300.

Poulton, S. W., Bekker, A., Cumming, V. M., Zerkle, A. L., Canfield, D. E., and Johnston, D. T., 2021, A 200-million-year delay in permanent atmospheric oxygenation: Nature, v. 592, no. 7853, p. 232-236.

Premo, W. R., and Van Schmus, W. R., 1989, Zircon geochronology of Precambrian rocks in southeastern Wyoming and northern Colorado, *in* Grambling, J. A., and Tewksbury, B. J., eds., Proterozoic Geology of the Southern Rocky Mountains, Volume 235, Geological Society of America Special Paper, p. 1-12.

Rasmussen, B., Bekker, A., and Fletcher, I. R., 2013, Correlation of Paleoproterozoic glaciations based on U–Pb zircon ages for tuff beds in the Transvaal and Huronian Supergroups: Earth and Planetary Science Letters, v. 382, p. 173-180.

Rasmussen, B., Blake, T. S., and Fletcher, I. R., 2005, U-Pb zircon age constraints on the Hamersley spherule beds: Evidence for a single 2.63 Ga Jeerinah-Carawine impact ejecta layer: Geology, v. 33, no. 9, p. 725-728.

Rasmussen, B., Fletcher, I. R., Muhling, J. R., Mueller, A. G., and Hall, G. C., 2007, Bushveld-aged fluid flow, peak metamorphism, and gold mobilization in the Witwatersrand basin, South Africa: Constraints from in situ SHRIMP U-Pb dating of monazite and xenotime: Geology, v. 35, no. 10, p. 931-934.

Redden, J. A., Peterman, Z. E., Zartman, R. E., and DeWitt, E., 1990, U-Th-Pb geochronology and preliminary interpretation of Precambrian tectonic events in the Black Hills, South Dakota, *in* Lewry, J. F., and Stauffer, M. R., eds., The Early Proterozoic Trans-Hudson Orogen of North America, Geological Association of Canada Special Paper 37, p. 229-251.

Schidlowski, M., and Todt, W., 1998, The Proterozoic Lomagundi carbonate province as paragon of a13C-enriched carbonate facies: Geology, radiometric age and geochemical significance: Chinese Science Bulletin, v. 43, no. Suppl 1, p. 114-114.

Schröder, S., Beukes, N. J., and Armstrong, R. A., 2016, Detrital zircon constraints on the tectonostratigraphy of the Paleoproterozoic Pretoria Group, South Africa: Precambrian Research, v. 278, p. 362-393.

Seymour, D. B., Thorne, A. M., and Blight, D. F., 1988, Wyloo Western Australia Geological Survey of Western Australia

Simonson, B. M., Schubel, K. A., and Hassler, S. W., 1993, Carbonate sedimentology of the early Precambrian Hamersley Group of Western Australia: Precambrian Research, v. 60, no. 1, p. 287-335.

Slomp, C. P., and Van Cappellen, P., 2007, The global marine phosphorus cycle: sensitivity to oceanic circulation: Biogeosciences, v. 4, no. 2, p. 155-171.

Smith, R. E., Perdrix, J. L., and Parks, T. C., 1982, Burial Metamorphism in the Hamersley Basin, Western Australia: Journal of Petrology, v. 23, no. 1, p. 75-102.

Souza, P. C., and Muller, G., 1984, Primeiras Estruturas Algais Comprovadas na Formac¸a˜o Gandarela,

Quadrila´tero Ferrı´fero: Revista Escola de Minas Ouro Preto, v. 2, p. 13-21.

Stanley, S. M., 2010, Relation of Phanerozoic stable isotope excursions to climate, bacterial metabolism, and major extinctions: Proceedings of the National Academy of Sciences, v. 107, no. 45, p. 19185-19189.

Stowe, C. W., 1978, Structure of the Lomagundi Group in the Sinoia area, Rhodesia: Special Publication of the Geological Society of South Africa, v. 4, p. 449-459.

Swart, Q. D., 1999, Carbonate rocks of the Paleoproterozoic Pretoria and Postmasburg Groups, Transvaal Supergroup [M.S. thesis: Rand Afrikaans University.

Swett, K., and Knoll, A. H., 1989, Marine pisolites from Upper Proterozoic carbonates of East Greenland and Spitsbergen: Sedimentology, v. 36, no. 1, p. 75-93.
Tennick, F. P., and Phaup, A. E., 1976, The geology of the country around Magondi, Lomagundi, Hartley and Gatooma Districts: Bulletin of the Geological Survey of Rhodes v. 314, p. 314.
Vallini, D. A., Cannon, W. F., and Schulz, K. J., 2006, Age constraints for Paleoproterozoic glaciation in the Lake Superior Region: detrital zircon and hydrothermal xenotime ages for the Chocolay Group, Marquette Range Supergroup: Canadian Journal of Earth Sciences, v. 43, no. 5, p. 571-591.
Walraven, F., 1997, Geochronology of the Rooiberg Group, Transvaal Supergroup, South Africa: University of the Witwatersrand.
Warke, M. R., 2017, Stratigraphic and geochemical framework of the Palaeoproterozoic rise in atmospheric oxygen: Transvaal Supergroup (South Africa) [Ph.D.: The University of Manchester
Weber, F., 1968, Une série précambrienne du Gabon: le Francevillien. Sédimentologie, géochimie, relations avec les gîtes minéraux associés, Persée-Portail des revues scientifiques en SHS, v. 1.
Woodhead, J. D., and Hergt, J. M., 1997, Application of the `double spike' technique to Pb-isotope geochronology: Chemical Geology, v. 138, no. 3, p. 311-321.
Young, G. M., 1983, Tectono-sedimentary history of Early Proterozoic rocks of the northern Great Lakes region, *in* Medaris Jr., L. G., ed., Early Proterozoic Geology of the Great Lake Region, Volume 160, Geological Society of America Memoirs.
